# Structure of the Receptor-Binding Carboxy-Terminal Domain of the Bacteriophage T5 L-Shaped Tail Fibre with and without Its Intra-Molecular Chaperone

**DOI:** 10.3390/v7122946

**Published:** 2015-12-08

**Authors:** Carmela Garcia-Doval, José R. Castón, Daniel Luque, Meritxell Granell, José M. Otero, Antonio L. Llamas-Saiz, Madalena Renouard, Pascale Boulanger, Mark J. van Raaij

**Affiliations:** 1 Departamento de Estructura de Macromoléculas, Centro Nacional de Biotecnología, Consejo Superior de Investigaciones Científicas (CNB-CSIC), calle Darwin 3, E-28049 Madrid, Spain; c.garcia@bioc.uzh.ch (C.G.-D.); jrcaston@cnb.csic.es (J.R.C.); dluque@cnb.csic.es (D.L.); meritxell.granell@cbs.cnrs.fr (M.G.); 2 Centro Nacional de Microbiología, ISCIII, Majadahonda, E-28220 Madrid, Spain; 3 Centro Singular de Investigación en Química Biolóxica e Materiais Moleculares, Universidade de Santiago de Compostela, E-15782 Santiago de Compostela, Spain; jose.otero@usc.es; 4 Unidade de Raios X, RIAIDT, Universidade de Santiago de Compostela, E-15782 Santiago de Compostela, Spain; antonio.llamas@usc.es; 5 Institute for Integrative Biology of the Cell (I2BC), CEA, CNRS, Univ Paris-Sud, Université Paris-Saclay, F-91198 Gif-sur-Yvette cedex, France; madalena.renouard@i2bc.paris-saclay.fr (M.R.); pascale.boulanger@i2bc.paris-saclay.fr (P.B.)

**Keywords:** bacterial viruses, *Caudovirales*, *Siphoviridae*, crystallography, infection, J0101

## Abstract

Bacteriophage T5, a *Siphovirus* belonging to the order *Caudovirales*, has a flexible, three-fold symmetric tail, to which three L-shaped fibres are attached. These fibres recognize oligo-mannose units on the bacterial cell surface prior to infection and are composed of homotrimers of the pb1 protein. Pb1 has 1396 amino acids, of which the carboxy-terminal 133 residues form a trimeric intra-molecular chaperone that is auto-proteolyzed after correct folding. The structure of a trimer of residues 970–1263 was determined by single anomalous dispersion phasing using incorporated selenomethionine residues and refined at 2.3 Å resolution using crystals grown from native, methionine-containing, protein. The protein inhibits phage infection by competition. The phage-distal receptor-binding domain resembles a bullet, with the walls formed by partially intertwined beta-sheets, conferring stability to the structure. The fold of the domain is novel and the topology unique to the pb1 structure. A site-directed mutant (Ser1264 to Ala), in which auto-proteolysis is impeded, was also produced, crystallized and its 2.5 Å structure solved by molecular replacement. The additional chaperone domain (residues 1263–1396) consists of a central trimeric alpha-helical coiled-coil flanked by a mixed alpha-beta domain. Three long beta-hairpin tentacles, one from each chaperone monomer, extend into long curved grooves of the bullet-shaped domain. The chaperone-containing mutant did not inhibit infection by competition.

## 1. Introduction

Bacteriophages are ubiquitous viruses of bacteria. They are important biological model systems and have been applied in detection and control of pathogenic bacteria [1,2]. More than 95% of all phages belong to the *Caudovirales* (tailed phages) [3]. This order is divided into three families, according to phage tail morphology: *Myoviridae*, *Podoviridae* and *Siphoviridae*. Around half of all known bacteriophages are *Siphoviruses* and have long, flexible, non-contractile tails. The well-studied *Siphovirus* T5 has a three-fold symmetric tail-tube, to the end of which three long, L-shaped fibres are attached [4,5]. These fibres are made up of a homotrimer of protein pb1 (pb stands for “protein band”). The gene encoding pb1 specifies 1396 amino acids, although the carboxy-terminal 133 residues are auto-proteolytically removed after correct trimerization and folding. It is thought that the trimer of these carboxy-terminal 133 residues functions as an intra-molecular chaperone [6,7].

The L-shaped tail fibres are responsible for the initial recognition of certain *Escherichia coli (E. coli)* host strains, binding to the O8- or O9-type O-antigen of the bacterial lipopolysaccharide (LPS) [6]. The structures of the O8 and O9 antigens have been characterized before [8,9]; the minimal region in common is a tri-mannose with alpha(1,2)-linkages. The pb1-LPS interaction is reversible, and, in fact, the presence of the fibres on the phage is not absolutely necessary, although they increase the adsorption rate by a factor of 15 [6,10]. In a secondary, irreversible, interaction, the central straight tail fibre interacts with the specific receptor of T5, the bacterial iron transporter protein FhuA. This interaction is mediated by the receptor binding protein pb5, which is located at the end of the straight fibre [11], and leads to release of the phage DNA into the host cell [12,13].

Here, we present the structure of the carboxy-terminal domain of the bacteriophage T5 L-shaped tail fibres, solved in the absence and presence of the intra-molecular chaperone domain and show that only the processed, mature protein can inhibit phage infection by competition.

## 2. Materials and Methods

### 2.1. Protein Expression, Purification, Crystallization and Stability Measurement

Production and crystallization of pb1(970–1263) was described before [14]. Upon expression and protein folding, the intra-molecular chaperone domain is auto-proteolytically cleaved off before purification and crystallization. To prevent auto-proteolytic cleavage and obtain a carboxy-terminal domain including the chaperone, the Ser1264-codon was mutated to code for Ala using the QuikChange Site-Directed Mutagenesis Kit (Agilent, Las Rozas, Madrid, Spain). The resulting expression plasmid was named pET28-pb1(970–1396)S1264A and yields pb1(970–1396), which was expressed and purified as described for the wild-type protein [14]. Crystallization trials for pb1(970–1396) were performed by vapor diffusion in sitting drop Compact Clover plates (Jena Biosciences, Jena, Germany) with 0.15 mL reservoirs and drops of 2 μL of protein solution mixed with 2 μL of reservoir solution. Lens-shaped crystals up to 0.15 mm long were obtained in drops where the reservoir contained 0.1 M sodium citrate pH 4.0 and 12.5% (*w*/*v*) PEG 2000 and were briefly soaked with the same solution including 20% glycerol before data collection.

Crystallization trials for native pb1(970–1263) in the presence of mannose were also performed (although no ligand was identified in the electron density). The purified protein (at 15 mg/mL in 20 mM Tris-HCl pH 8.5) was incubated in the presence of 2 mM mannose for 2 h at room temperature. Crystals were obtained in a drop where the reservoir contained 0.1 M Tris-HCl pH 8.5, 8% (*w*/*v*) PEG 4000, 10 mM iron(III) chloride and 20% (*v*/*v*) glycerol and were rapidly cryo-cooled directly in liquid nitrogen.

Thermal denaturation assays were performed in an iCycler iQ PCR Thermal Cycler (Bio-Rad, Hercules, CA, USA) in the presence of SYPRO Orange (Life Technologies SA, Madrid, Spain). Reaction volumes of 30 μL were prepared in 200 μL capped PCR tubes with 30 μg of protein and 1× SYPRO Orange from the supplied 5000× stock solution. Samples were heated from 4 °C to 94 °C with a ramp rate of 1 °C/min; the fluorescence was measured at every 0.5 °C increment.

### 2.2. Phage Infection Inhibition

The O-antigen dependent bacteriophage T5oad mutant [15] was used to test for inhibition of phage T5 adsorption by the C-terminal domain of pb1. Due to a mutation in the gene encoding the receptor binding protein pb5, this mutant binds the outer membrane protein receptor FhuA with a low affinity and, as a consequence, successful infection is strictly dependent on the interaction between the L-shaped tail fibers and polymannose O-antigen of *E. coli* F. T5oad was amplified on the *E. coli* F strain and purified by centrifugation on cesium chloride gradients [16]. *E. coli* F bacteria were harvested in the mid-log phase, washed in phosphate-buffered saline and resuspended at a cell density of 1 × 10^9^/mL in T5-TBS buffer (10 mM Tris-HCl pH 7.2, 100 mM sodium chloride, 1 mM calcium chloride and 1 mM magnesium chloride). Aliquots (0.05 mL) of cell suspension were incubated for 20 min at 0 °C with concentrations (0.015 to 1.5 μM) of purified pb1(970–1263) or pb1(970–1396). Estimating that a bacterial cell contains about 10^6^ LPS molecules [17], this corresponds to a ratio of trimeric pb1 to LPS of 0.01 to 1. Phage T5oad mutant was then added at a multiplicity of infection of 1 and incubation was continued for 15 min at 37 °C. The mixture was then diluted 500-fold into ice cold T5-TBS buffer and centrifuged at 6000 × g for 6 min. The amount of unadsorbed phage particles remaining in the supernatant was determined by titration on *E. coli* F. In these experimental conditions, we confirmed that adsorption of T5oad to *E. coli* B, which has a semi-rough LPS, lacking the O-specific chain, was less than 1%. The presence of 8.5 μM of bovine serum albumin did not affect the adsorption of T5oad to *E. coli* F.

### 2.3. Crystallographic Structure Solution

Data collection for selenomethionine-modified pb1(970–1263) has been described and statistics reported previously [14]. Data for native pb1(970–1396)S1264A were collected at beamline BL13-XALOC [18] of the ALBA-CELLS synchrotron (Barcelona, Spain), using a Pilatus 6M detector (Dectris, Baden, Switzerland). Data were integrated with MOSFLM [19] and scaled with AIMLESS [20]. The structure of selenomethionine-modified pb1(970–1263) was solved by single-wavelength anomalous dispersion using the AUTOSHARP procedure [21]: fifteen selenium atoms were localized with SHELX [22], phases were refined and solvent flattening was performed with SOLOMON [23]. Molecular replacement was performed with PHASER [24]. Automatic model building was performed with PHENIX [25] and model completion, adjustments and adding solvent molecules were performed with COOT [26]. Refinement was performed with REFMAC [27]. Model validation was done with MOLPROBITY [28]. Protein interaction interfaces were assessed with PISA [29] and homology to known structures with DALI [30]. Programs from the CCP4 suite [31] were also used. Figure 2, Figure 4, Figure 5 and Figure 6 were prepared with PYMOL [32]; Figure 7 was prepared with UCSF CHIMERA [33]. Coordinates and structure factors have been submitted to the protein structure database under codes 4UW7 for selemethionine-containing pb1(970–1263), 5AQ5 for native methionine-containing pb1(970–1263) and 4UW8 for pb1(970–1396), respectively.

### 2.4. Electron Microscopy of pb1(970–1263) and pb1(970–1396)

Samples (5 μL) of purified pb1(970–1293) and pb1(970–1396) proteins were applied to glow-discharged carbon-coated grids and negatively stained with 2% uranyl acetate solution. Images were recorded on a FEI Eagle 4k CCD camera in low-dose conditions with a Tecnai G2 electron microscope (FEI, Hillsboro, OR, USA) operating at 200 kV. Frames were recorded at a detector magnification of 67,873× (2.21 Å/pixel sampling rate). Electron microscopy of bacteriophage T5 tails was described before [14].

Image processing was performed using XMIPP [34] and defocus was determined with CTFFIND3 [35]. For bacteriophage T5 tail samples, we selected 1129 images of the distal part of the fibre; classification was used to only select straight distal parts, as described [14]. The XMIPP manual particle picking interface was used to select 2006 and 1375 individual images of pb1(970–1263) and pb1(970–1396) assemblies, respectively. After alignment and classification using maximum likelihood routines [36], we selected 444 straight rod images of the tail fibre to obtain a two-dimensional average image. The complete datasets of manually picked particles of pb1(970–1293) and pb1(970–1396) were used to obtain their respective average images (no classification was used). The resulting images were rotationally averaged around their longitudinal axis to render averaged three-dimensional volumes. The UCSF CHIMERA fit routine was used to computationally dock the X-ray structures of pb1(970–1293) in the fibre and in pb1(970–1293) averaged volumes, and that of pb1(970–1396) in the pb1(970–1396) averaged map, with correlation coefficients of 93%, 95% and 96%, respectively.

## 3. Results

### 3.1. Protein Expression, Purification and Crystallization

The proteins pb1(794–1263) and pb1(970–1263), corresponding to two carboxy-terminal (distal) fragments of the L-shaped tail fibres of different size, were expressed, purified and crystallized as described before [14]. A single point mutation (T to G), mutating the codon of the corresponding serine in pb1 (Ser1264) to code for alanine, was introduced into the plasmid pET-28-pb1(970–1396). Expression of the mutated gene in *E. coli* cells led to production of the non-proteolyzed version of the protein, which we call pb1(970–1396). Substitution of a conserved serine to alanine was previously described to inhibit the auto-proteolytic cleavage of a similar intra-molecular chaperone domain [7,37]. Pb1(970–1396) was purified by metal affinity chromatography and anion exchange chromatography and crystallized from poly-ethylene glycol solutions at pH 4.

### 3.2. Phage Infection Inhibition

**Figure 1:**
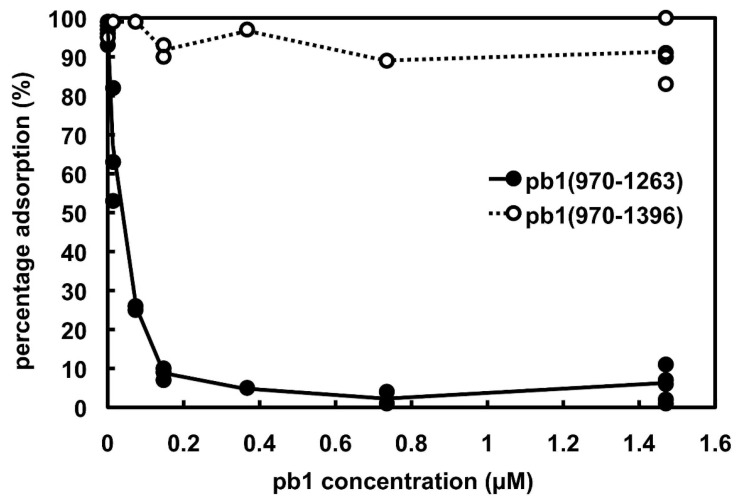
Inhibition of L-shaped tail-fibre dependent bacteriophage T5oad mutant by purified pb1 proteins. *Escherichia coli* F (5 × 10^7^ cells in 0.05 mL) were incubated with pb1(970–1263), the carboxy-terminal domain of the mature protein and pb1(970–1396), the carboxy-terminal domain of the uncleaved mutant protein at the concentrations shown and un-adsorbed phages were titrated by plaque counting. Concentrations are of the trimeric proteins.

We expected the carboxy-terminal end of the L-shaped tail fibres to be the specific domain that binds to the O-antigen of the LPS. To confirm this, we measured the inhibition of phage T5oad adsorption by the carboxy-terminal domain of pb1. Unlike wild-type T5, the T5oad mutant is strictly dependent on the binding of the L-shaped fibers to polymannose O antigen of *E. coli* F to successfully infect its host [15]. Thus, addition of the carboxy-terminal domain of pb1 to *E. coli* F bacteria is expected to inhibit infection with T5oad. The inhibitory effects of purified pb1(970–1263) and pb1(970–1396) are shown in Figure 1. More than 90% inhibition was measured when T5oad was incubated with pb1(970–1263) at concentrations ranging from 0.015 to 1.5 μM of the trimeric proteins, while pb1(970–1396) does not significantly inhibit T5oad adsorption at the same concentrations. These data indicate that the pb1(970–1263) domain likely binds to the O-antigen receptor. As the presence of the intra-molecular chaperone domain prevents inhibition of T5 adsorption, we propose that the binding site for the O-antigen is located at the terminal end the L-shaped tail fibre and is unmasked after cleavage of the chaperone domain.

### 3.3. Structure Solution

**Figure 2:**
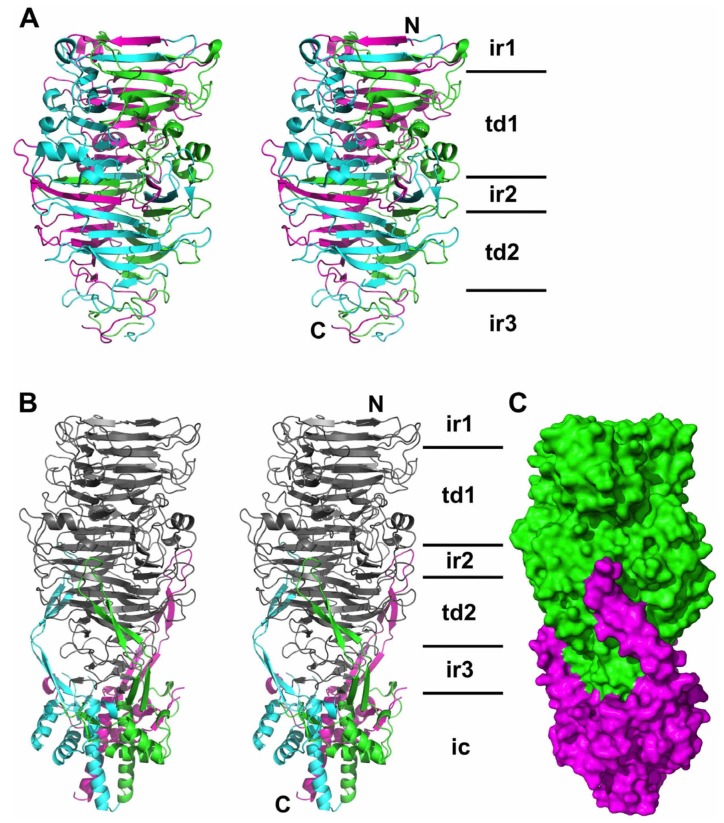
Structure of the carboxy-terminal domain of the bacteriophage T5 L-shaped tail fibre. (**A**) Overall side stereo-view of pb1(970–1263) in cartoon representation. Monomers are coloured differently (chain A green, chain B blue, chain C magenta). The first interdigitated region (ir1), first triangular domain (td1), second interdigitated region (ir2), second triangular domain (td2) and third interdigitated region (ir3) are indicated (interdigitated regions and triangular domains have been described in phage tailspike structures before, see text). The amino-terminal (N) and carboxy-terminal ends of the magenta subunits are indicated; (**B**) Overall side stereo-view of pb1(970–1396) in the same orientation as part A. Pb1(970–1263) is shown in gray, the monomers of the intra-molecular chaperone domain (ic) coloured differently (chain A green, chain B blue, chain C magenta); (**C**) Space-filling diagram in the same orientation as in part B. The carboxy-terminal part of the protein is coloured green, the intra-molecular chaperone domain in magenta.

A dataset to 2.5 Å resolution of the seleno-methionine derivative of pb1(970–1263) [14] collected at the selenium peak was used as input for the phasing procedure. Fifteen selenium atoms were located as expected; the asymmetric unit of the crystal contains one trimer, and each pb1(970–1263) monomer has five methionine residues. After solvent flattening, an initial model was produced by automatic tracing, which was improved manually. The resulting model was used to phase the structure of pb1(970–1396) by molecular replacement. Three trimers were located in the asymmetric unit, and the intra-molecular chaperone region could be constructed by a combination of automated and manual building in the calculated electron density. Density for residues 1297–1303 is missing in each of the nine monomers; these residues likely form a disordered loop. The structures of pb1(970–1263) and pb1(970–1396) (Figure 2) were refined to R-factors of 21.3% and 18.5% and free R-factors of 26.6% and 21.3%, respectively, with good geometry and few residues in unlikely regions of the Ramachandran plot (Table 1). Interpretable density was present neither for residues 970–987, nor for the amino-terminal purification tag. The data collected by Garcia-Doval *et al.* [14] for native pb1(794–1263) and pb1(970–1263) was also used in molecular replacement experiments, but in the case of pb1(794–1263), no density for extra N-terminal residues was observed and the remaining part could not be refined to satisfactory R-factors. In the case of pb1(970–1263), no satisfactory solution could be found by molecular replacement. However, we did collect data from a new crystal form of native methionine-containing protein, and refined this structure at 2.3 Å resolution (Table 1).

When the monomers in the pb1(970–1263) structure are superposed, they align with a root mean square difference (r.m.s.d.) of only 0.3 Å over 269 Cα-atoms; even loop residues superpose very well. The same is true for the pb1(970–1396) structure and here the r.m.s.d. is between 0.3 and 0.5 Å when 392–394 Cα-atoms are superposed. When the trimer of pb1(970–1263) is superposed onto the equivalent domain in pb1(970–1396), the r.m.s.d. is only 0.5 Å for 824 superposed Cα-atoms.

**Table 1:** Data collection, crystallographic phase determination and refinement statistics.

Protein	SeMet-pb1(970–1263) ^1^	pb1(970–1263)	pb1(970–1396)
*Data collection*			
Wavelength (Å)	0.9797	0.9795	1.0972
Space group	*C*2	*P*1	*C*2
Cell edges (a, b, c, Å)	227.4, 58.2, 69.9	86.6, 95.0, 127.7	160.9, 99.3, 286.2
Cell angles (α, β, γ, °)	90.0, 98.8, 90.0	68.2, 70.2, 83.6	90.0, 91.5, 90.0
Resolution range (Å)	56.2–2.52 (2.59–2.52) ^2^	23.0–2.30 (2.42–2.30)	95.4–2.52 (2.65–2.52)
Unique reflections	30585 (2237)	144381 (20958)	150772 (21539)
Multiplicity	6.3 (6.5)	2.4 (2.4)	4.1 (3.8)
Completeness	0.968 (0.958)	0.913 (0.905)	0.988 (0.972)
I/sigma(I)	11.4 (2.3)	7.6 (3.0)	6.3 (2.5)
Rsym ^3^	0.100 (0.644)	0.080 (0.209)	0.140 (0.433)
CC Imean	0.996 (0.869)	0.995 (0.982)	0.979 (0.784)
*Phase determination*			
Resolution range (Å)	56.2–2.52 (2.59–2.52) ^2^		
Anomalous correlation coefficient	0.508 (0.059)		
Correlation coefficient (all/weak) ^3^	41.20/24.01		
Patterson figure of merit ^4^	12.07		
Correlation coefficient (E) ^4^	0.294		
R-Cullis ^5^	0.780		
Phasing power ^5^	1.160		
Figure of merit ^5^	0.327		
Solvent flattening ^6^	46.4% solvent		
R-factor before/after density modification ^6^	0.5000/0.1820		
Correlation on |E|^2^ before/after dens. mod. ^6^	0.2850/0.6945		
Hand score (original/inverted) ^6,7^	0.3176/0.4066		
*Refinement*			
Resolution range used (Å)	56.2–2.52 (2.66–2.52) ^2^	23.0–2.30 (2.42–2.30)	95.4–2.52 (2.65–2.52)
Number of reflections used	28484 (4242)	140892 (20160)	148385 (21161)
Number of reflections used for R_free_	2035 (201)	2906 (467)	2061 (312)
R-factor ^8^	0.213 (0.33)	0.223 (0.28)	0.185 (0.28)
R-free	0.266 (0.38)	0.252 (0.31)	0.213 (0.29)
Atoms (protein/water/other solvent)	6144/109/18	24469/890/0	27591/936/117
Ramachandran statistics ^9^	0.931/0.996	0.972/1.000	0.976/0.999
R.m.s.d. ^10^ (bonds, Å/angles, °)	0.012/1.4	0.009/1.3	0.010/1.4
PDB code	4UW7	5AQ5	4UW8

^1^ Data collection statistics, but not phase determination or refinement statistics, for SeMet-pb1(970–1263) were reported before [14]; ^2^ Values in parentheses are for the highest resolution bin, where applicable; ^3^ R_sym_ = Σ_h_Σ_i_|I_hi_ − <I_h_>| / Σ_h_Σ_i_|I_hi_|, where I_hi_ is the intensity of the *i*th measurement of the same reflection and <I_h_> is the mean observed intensity for that reflection; ^4^ Found by SHELXD; ^5^ Calculated with AUTOSHARP; ^6^ According to SOLOMON; ^7^ Correlation on |E|^2^ / contrast; ^8^ R = Σ||*Fobs(hkl)* | − |*Fcalc(hkl)* || / Σ|*Fobs(hkl)* |; ^9^ Determined with MOLPROBITY. The fractions are indicated of residues in favoured and allowed regions of the Ramachandran plot, respectively; ^10^ Provided by REFMAC (r.m.s.d. is the root mean square deviation).

### 3.4. The Carboxy-Terminal Domain of the Mature Fibre

**Figure 3:**
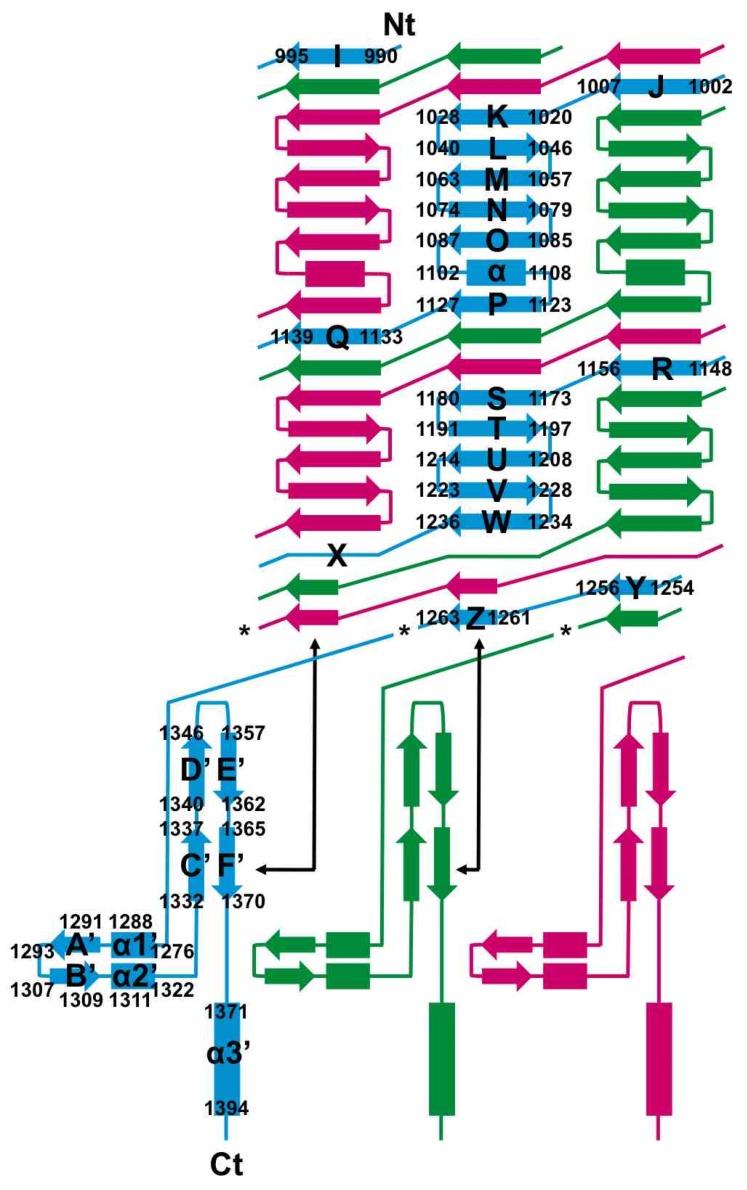
Topology diagram of pb1(970–1396). Beta-strands are represented as arrows and alpha helices as rectangles. Secondary structure elements are labelled for the cyan monomer. Asterisks indicate the location of the scissile Ser1264. The two-way arrows indicate the interaction completing the YZF′C′ beta-sheet. The beginning and end of each secondary structure element is labelled with the residue number.

The structure of pb1(970–1263) resembles a bullet (Figure 2A). We divided the structure in five different regions and have named the secondary structure elements as shown in Figure 3 (based on the podovirus P22 tailspike (PDB entry 1TSP) [38]). The amino-terminal part (amino acids 989–1009, beta-strands I and J) forms a first “interdigitated” region (ir1). In this region, the beta-strands of the three monomers wrap around each other to form a triple beta-helix [38,39]. Next, a first “triangular domain” (residues 1010–1129, beta-strands K through P) is present. Here, three concave beta-sheets form a beta-prism (td1). It is composed of the anti-parallel beta strands K through O, with strand P oriented parallel to strand O and connected to it by a long loop containing an alpha-helix.

**Figure 4:**
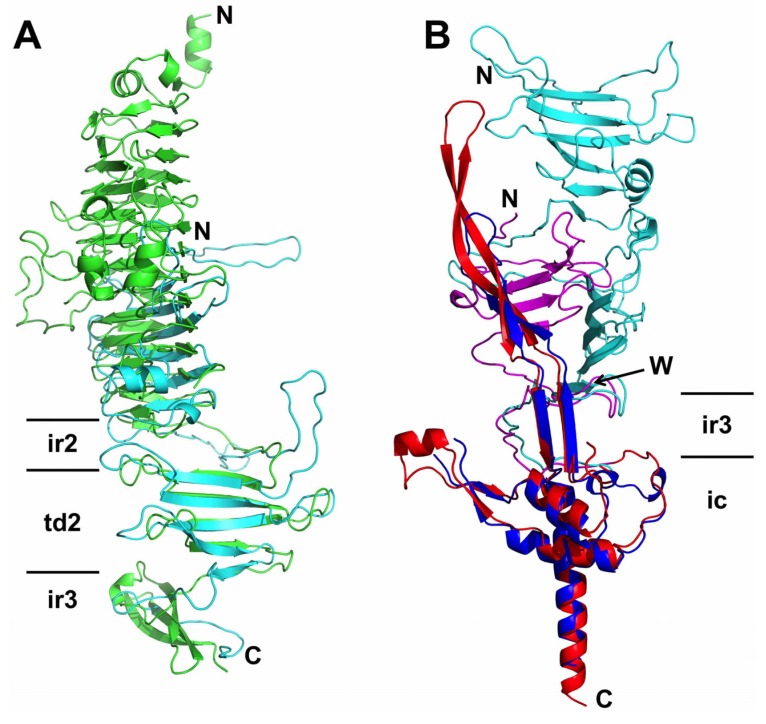
Structural superposition of the phage T5 L-shaped fibre domains with homologous structures. (**A**) Superposition of a monomer pb1(970–1263) in cyan and P22 tailspike protein (113–666) (PDB 1TSP) in green; (**B**) Pb1(970–1396) in cyan with its intra-molecular chaperone domain in blue superposed onto the phage K1F endosialidase (PDB 3GW6) in magenta, with its chaperone domain in red. Strand W of the pb1(970–1396) structure is signalled with an arrow; N- and C-termini of the proteins are indicated. Domains described in the text are indicated using the same nomenclature as in Figure 2.

There are no proteins in the protein data bank (PDB) with the same topology as the pb1 bullet-shaped domain, although the topologies of some bacteriophage tailspikes, such as the podovirus P22 tailspike (PDB entry 1TSP) [38], partially overlap. Phage P22 tailspike has a beta-helical domain, an interdigitated region, a triangular beta-prism domain and a second interdigitated region (called caudal fin). Of these, the triangular beta-prism is the most similar to the second triangular domain of pb1 and has the same topology (Figure 4A). The P22 tailspike has endo-rhamnosidase activity; however, pb1 has no known enzymatic activity. Consequently, the catalytic residues of the P22 tailspike are not conserved in pb1.

The second interdigitated region ir2 (residues 1130–1160, beta-strands Q and R) again forms a short triple beta-helix, with the beta strands in parallel disposition. This is followed by a second triangular domain td2 (residues 1161–1238). Like the first triangular domain, this domain is also a beta-prism formed by three concave beta-sheets, each containing five anti-parallel beta-strands (S through W). At the distal end of the mature protein, a third interdigitated region (ir3) is located, made up of residues 1239–1263, which form a tapered triple-helical structure. The taper makes the end of the structure pointed. In the mature, chaperone-free structure, the third interdigitated region does not contain any regular beta-structure, although there are many inter-chain main-chain hydrogen bonds. In the structure with the chaperone, two short beta-strands are present in the third interdigitated region, forming a four-stranded beta-sheet with the C′ and F′ beta-strands of the chaperone domain (Figure 2B and Figure 3). Generally, the loops connecting the beta-strands in the structure are long and elaborate, especially the aforementioned OP-loop and the RS-loop, which is bent back as a hairpin towards the amino-terminus.

### 3.5. The Intra-Molecular Chaperone Domain

Serine 1264, the residue responsible for the auto-proteolytic cleavage of the carboxy-terminal domain from the bullet-shaped domain, is located between the bullet-shaped and intra-molecular chaperone domains. In the structure where Ser1264 has been substituted with alanine, the chain continues with a loop (residues 1265–1276) winding around the other three, subsequently forming a hairpin (residues 1277–1322). This first hairpin contains two alpha-helices and two beta-strands (A' and B') and is oriented side-ways, nestled under the aforementioned loop of amino acids 1265–1276. Three 50 Å-long hairpins (residues 1332–1371 from each monomer, containing beta-strands C' through F', protrude upwards and embrace the preceding bullet-shaped domain). The intra-molecular chaperone domain (ic) ends in a central triple-helical coiled-coil, to which each monomer contributes their carboxy-terminal alpha-helix (formed by residues 1371–1394).

The structure of the intra-molecular chaperone domain shows similarity to the equivalent domain of the *E. coli* phage K1F endosialidase (PDB entry 3GW6) [37] and can be superposed to an r.m.s.d. of 2.4 Å with 122 superposed Cα atoms, despite a sequence identity of only 18%. When monomers of the two structures are superposed (Figure 4B), it is clear that they have the same fold and topology, with all the secondary structure elements conserved. The beta-hairpin tentacles in our structure are about 20 Å shorter than in the K1F endosialidase, while the A'B'-loop, that is partially disordered in our structure, contains an alpha-helix in the structure of the K1F endosialidase chaperone domain. The similarity between pb1 and the K1F endosialidase extends into the triple-helical tapered domain and the last strand of the second triangular domain (strand W), down to residue 1232 (Figure 4B; r.m.s.d. of 3.3 Å with 188 Cα-atoms superposed). However, where in pb1 the VW-loop folds back, in the K1F endosialidase the triple beta-helix continues for one more strand. When the quaternary structures are compared, they are still similar, with the parallel beta-sheet permuted onto another side of the trimer. The structure of the intra-molecular chaperone domain of the neck appendage protein of *Bacillus* phage GA-1 (PDB entry 3GUD) [37] is also very similar to the pb1 chaperone, while the structure of the neck appendage protein chaperone domain from *Bacillus* phage phi29 (PDB entry 3SUC) [40] reveals more differences: its body has a beta-stranded fold, is not elongated as in the other chaperones and the beta-hairpin tentacles are much shorter.

### 3.6. Trimer Stability

Analysis of the quaternary structure of both pb1(970–1263) and pb1(970–1396) shows that more than half of each surface monomer is buried in the trimer: 13 × 10^3^ Å^2^ of 23 × 10^3^ Å^2^ for pb1(970–1263) and 19 × 10^3^ Å^2^ of 35 × 10^3^ Å^2^ for pb1(970–1396). The estimated free energy gains upon trimerization are 285 and 380 kcal/mol for pb1(970–1263) and pb1(970–1396), respectively. Thermofluor unfolding experiments were performed on both pb1(970–1263) and pb1(970–1396). In these experiments, a sample of the protein is incubated in the presence of a hydrophobic fluorescent dye and the temperature is slowly raised. When the protein unfolds, the dye increasingly binds to hydrophobic residues of the protein and the fluorescence increases. The experiments showed that pb1(970–1263) unfolds at 62 to 64 °C, while pb1(970–1396) unfolds between 64 and 65 °C, indicative of stable proteins.

**Figure 5:**
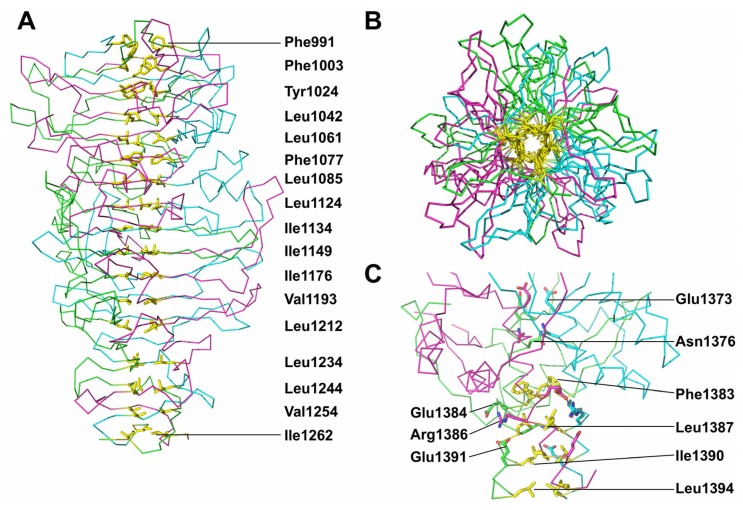
Inter-monomer interactions in the bacteriophage T5 L-shaped fibre trimer. (**A**) Side view of the C-terminal fibre domain, with the residues contributing to the central hydrophobic core shown in yellow and labelled on the right; (**B**) Top view of A, illustrating how the aromatic and hydrophobic residues contribute to the core; (**C**) Central interactions in the chaperone domain. Hydrophobic side-chains are shown in yellow and other residues in the same colour as the protein chain they belong to.

The extensive intertwining of the monomers in the trimer and close association in other regions leads to the formation of numerous hydrogen bonds, van der Waals interactions and salt bridges (there are 16 putative inter-monomer salt bridges for pb1(970–1263) and 25 for pb1(970–1396)). The central, three-fold symmetric, longitudinal cavity between the three monomers is surrounded by hydrophobic and aromatic residues, for example Ile1134, Ile1149, Phe1044, Phe1077, Phe1174 and Trp1226 (Figure 5A,B). The interdigitated regions may be involved in the stabilization of the structure, keeping the three chains linked together as a molecular clamp [41].

In the chaperone, the bottom part of the centre of the chaperone coiled-coil is also hydrophobic, with Phe1383, Leu1387, Ile1390 and Leu1394 contributing to the core (Figure 5C). The top part is more hydrophilic, with Glu1373 and Asn1376 also pointing towards the centre, interacting with its equivalents from the two other monomers. The three Arg1386 residues point outwards from the coiled-coil but project their guanidine groups to between Glu1384 and Glu1391 of a neighbouring monomer. These hydrophilic interactions and salt bridges, apart from contributing to the stability of the trimer, may ensure correct registration (*i.e.*, avoid staggered interactions between the three monomers). The interactions also suggest that the fibre-distal part of the chaperone may be more rigid, and the fibre-proximal part more flexible, facilitating interaction with the fibre.

Although the interface between the bullet-shaped domain and the intra-molecular chaperone domain appears extensive (Figure 2C), it is only 3 × 10^3^ Å^2^ large and the association is accompanied by a free energy gain of just −12 kcal/mol. The interface contains almost exclusively hydrophilic interactions. Salt bridges are formed by the Glu1355 residues from the top of the tentacle with Arg1150 and Arg1222 projecting from the sides of the bullet-shaped domain. These residues are from three different monomers. After cleavage of the three Thr1263-Ser1264 peptide bonds, the chaperone must be released; therefore, the interaction between the bullet-shaped domain and intra-molecular chaperone domain must not be overly strong.

### 3.7. The Catalytic Site

**Figure 6:**
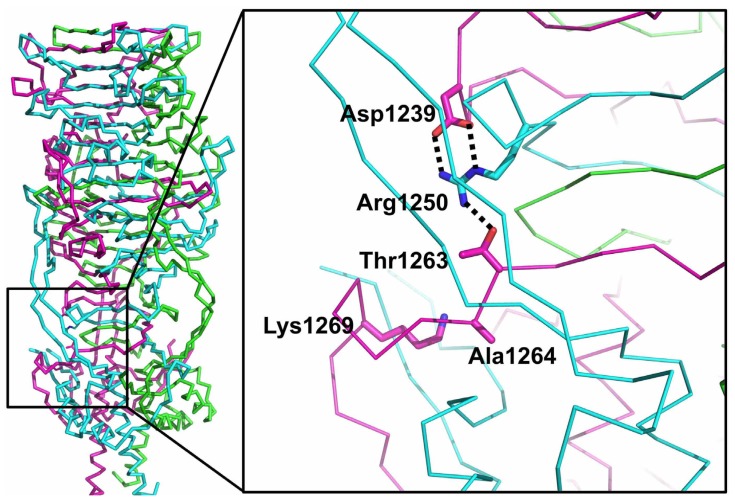
Residues proposed to be involved in the proteolytic cleavage reaction. The location of the guanidine group of Arg1250 of a neighbouring protein chain between the side-chains of Asp1239 and Thr1263 is shown. The catalytic Ser1264 would be where Ala1264 is in the structure of the uncleaved mutant protein. Lys1269 is also highlighted.

For the intra-molecular chaperone of the endosialidase of *E. coli* phage K1F, a mechanism has been proposed for the auto-proteolytic reaction [37,42]. All the residues involved in this proposed mechanism are conserved in the phage T5 L-shaped tail fibre sequence and in very similar positions in the structure, making it likely the reaction mechanism is the same. Transposing the mechanism to the L-shaped tail fibre, Arg1250 of a neighbouring subunit of the third interdigitated region acts as a sensor for correct folding, positioning its positively charged guanidine group between Asp1239 and the side chain carbonyl of Thr1263, forming the necessary oxy-anion hole for stabilizing the transition state (Figure 6). A kink in the main chain at the position of Thr1263 places the hydroxyl oxygen of the scissile Ser1264 near the amine group of Lys1269, activating it for the cleavage reaction. The involvement of Arg1250 of a neighbouring monomer makes the correct formation of the triple beta-helix fold a requirement for the auto-proteolytic cleavage to proceed. In the mature fibre, we do not observe density for the catalytic Ser1264, suggesting that this residue is removed with the intramolecular chaperone domain upon maturation of the fibre. The same was observed for the K1F tailspike [37,42], while in the phi29 appendage the serine was modelled as the last residue of the mature protein, albeit with less convincing density [40].

### 3.8. Electron Microscopy of Phage T5 Tail and Distal Pb1 Assemblies

**Figure 7:**
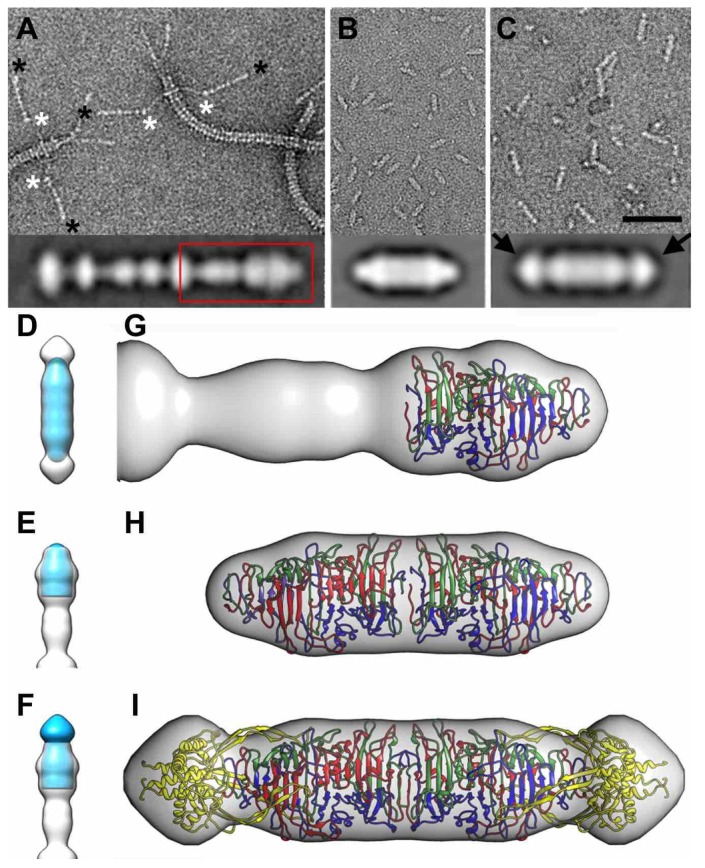
Electron microscopy of the bacteriophage T5 tail and expressed pb1 distal domains. (**A**) Negatively stained T5 tails, in which the some of the distal ends of the L-shaped fibres are indicated with black asterisks and the apparently flexible hinge, or kink, with white asterisks. The scale bar in (C) corresponds to 50 nm; parts (A) and (B) are at the same scale; (**B**) EM of expressed and purified pb1(970–1263); (**C**) EM of expressed and purified pb1(970–1396), *i.e.*, including the carboxy-terminal intra-molecular chaperone domain. Two-dimensional averages of side views of each T5 tail assembly are shown (bottom insets). The red rectangle highlights the most distal region of the tail fibre shown in parts (E), (F) and (G). Both types of purified protein associate into tail-to-tail dimers of trimers. The intra-molecular chaperone domain is clearly present on the ends of the protein in the right panel, as indicated by arrows; (**D**) Three-dimensional model of pb1(970–1263) (blue) superimposed on the three-dimensional model of pb1(970–1396) (transparent); (**E**) Three-dimensional half-model of pb1(970–1263) (blue, cropped at the longitudinal midpoint) superimposed on the three-dimensional model of the end of the L-shaped tail fibre end (transparent); (**F**) Three-dimensional half-model of pb1(970–1396) (blue, cropped at the longitudinal midpoint) superimposed on the three-dimensional model of the end of the L-shaped tail fibre end (transparent); (**G**) Three-dimensional model of the most distal region of the L-shaped tail fibre distal domain with the trimeric pb1(970–1263) atomic model docked at the tip; (**H**,**I**) Three-dimensional models of the trimer-dimers of pb1(970–1263) (H) and pb1(970–1396) (I) with their corresponding atomic models docked.

Purified T5 tails were analyzed by negative stain electron microscopy (Figure 7A) as reported before [14]; pb1(970–1263) and pb1(970–1396) assemblies were analyzed in the same way (Figure 7B,C). Averaged side views (Figure 7A–C, insets) were used to calculate a three-dimensional model by applying rotational symmetry over the longitudinal axes, as described [14]. The volumes obtained for pb1(970–1263) and pb1(970–1396), about 18 and 25 nm long, respectively, correspond to dimers of the trimeric protein fragments, interacting tail-to-tail through their amino-termini. Notably, this interaction was also observed in the crystal packing of both pb1(970–1263) and pb1(970–1396). Superimposition of the truncated pb1 assembly volumes onto the intact L-shaped tail distal domain volume showed that these structures matched well, indicating that the truncation did not alter the T5 tail fibre tip structures (Figure 7D–F); the additional volume of the intra-molecular chaperone flower bud-shaped domain was located at the tips of the dimeric pb1(970–1396) (Figure 7C, arrows). Docking analysis of pb1(970–1263) and pb1(970–1396) into their electron microscopy maps, as well as that of the T5 tail fibre, confirmed good matching (correlation coefficients of 93%–96%), with no notable structural alterations (Figure 7G–I). It also confirmed the tail-to-tail dimeric nature of pb1(970–1263) and pb1(970–1396).

## 4. Discussion

Like other bacteriophage fibres and tailspikes [38,39,40,43,44], the bullet-shaped distal domain of pb1 is mainly beta-structured. It is likely the structure down to the kink (*i.e.*, between the white and black asterisks in Figure 7A) also has beta-structure, while the thin proximal domain of pb1 may have a collagen fold [14].

Elongated trimeric beta-structured proteins have evolved different strategies to fold correctly. Some fold endogenously when expressed in bacteria, like the phage P22 tailspike [45] and the phage T7 fibre [46]. Others need one or more specialized chaperone proteins, like the fibre proteins gp12, gp34 and gp37 of bacteriophage T4 [47,48,49,50]. The bacteriophage T5 L-shaped tail fibre uses an intra-molecular chaperone to fold, which is then auto-proteolytically released to yield the mature protein. This mechanism is the same as in several bacteriophage endosialidases, capsule depolymerases and appendage proteins, such as *E. coli* phage K1F [37], *Bacillus* phages GA-1 [37] and phi29 [40] and presumably *E. coli* phages K1E, 63-D, K1–5, K5, *Bacillus* phages PZA, B103 and *Zymomonas mobilis* phage ZM4 [7,51]. Interestingly, the carboxy-terminal fin of the phage P22 tailspike has also been proposed to function as an auto-chaperone domain [52], although it is not cleaved after folding.

In phage T5 pb1, the folding may start from the distal part of the chaperone with the association of the three helices of the short triple coiled-coil. The chaperone domains fold and the upward-pointing tentacles are formed. These tentacles then interact with the bullet-shaped domain of the fibre, confining the chains of the bullet-shaped domain while they fold. Once the carboxy-terminal triple-helix is correctly formed, proteolysis can take place as described above [37,42], leading to release of the chaperone domain.

Bacteriophage T5 binds to the polymannose O-antigens of *Escherichia coli* LPS. The minimal unit described to bind to T5 fibre is a tri-mannoside [6]. Many proteins bind to carbohydrates through aromatic residues [53], but aromatic residues do not cluster on the surface of our pb1(970–1263) structure; therefore, we can currently not draw any conclusions on where the oligo-mannose units might bind to pb1. An attractive hypothesis would be a receptor-binding site in the groove that becomes exposed upon intra-molecular chaperone cleavage, and this is consistent with the results of the phage infection inhibition assays by competition with pb1(970–1263) but not by the chaperone-containing pb1(970–1396). Future site-directed mutagenesis and co-crystallization studies should lead to more insight into the exact location and nature of the receptor-binding site.

## 5. Conclusions

We have solved the structure of the carboxy-terminal domain of the *Siphoviridae* phage T5 tail fibre, with and without its carboxy-terminal intra-molecular chaperone domain. The structures reveal an extensively intertwined, beta-structured receptor-binding domain and an alpha/beta-structured intra-molecular chaperone domain, with long beta-hairpin fingers enveloping its folded substrate. Once receptor-binding sites have been identified, the structure may form the basis of rational design of phage-based fibres for specific recognition of target bacteria, together with the structures of the receptor-binding domains of fibres of *Myoviridae* phages [43,54] and *Podoviridae* phages [44].
